# Promoting safety of underground machinery operators through participatory ergonomics and fuzzy model analysis to foster sustainable mining practices

**DOI:** 10.1038/s41598-024-67375-1

**Published:** 2024-07-15

**Authors:** Vikram Sakinala, P. S. Paul, Yewuhalashet Fissha

**Affiliations:** 1 Department of Mining Engineering, Indian Institute of Technology (Indian School of Mines), Dhanbad, 826004 India; 2 Department of Mining Engineering, Aksum University, Aksum, 7080 Tigray, Ethiopia

**Keywords:** Fuzzy logic model, Muscular skeletal disorders, Participatory ergonomics, Sustainable mining, Work posture assessment, Work process assessment, Work tool assessment, Engineering, Sustainability, Health occupations

## Abstract

One of the most vital parameters to achieve sustainability in any field is encompassing the Occupational Health and Safety (OHS) of the workers. In mining industry where heavy earth moving machineries are largely employed, ergonomic hazards turn out to be significant OHS hazards causing Musculoskeletal Disorders (MSDs) in the operators. Nevertheless, the Indian mining industry lacks a comprehensive technique of OHS risk assessment, especially for ergonomic hazards that cause MSDs. This research appraises ergonomic hazards and develops Fuzzy Musculoskeletal-disorders Index (FMI) model to evaluate ergonomic-related MSDs. Work process and work tool ergonomic risk factors were identified through literature review and directives recommended by experts. Work posture was evaluated using RULA. The data-collecting approach was implemented using participatory ergonomic and design science principles. The FMI results show average MSDs score of 3.69, indicating high to extremely high risk. Surface plots show that combined work tool and work process was the most sensitive factors to MSDs risk compared to other two combinations. A two-sample *t*-test validated the FMI. The findings should help safety experts and managers develop effective OHS management plans and programmes for the sustainability of Indian mining industry.

## Introduction

Safety in mining operations is fundamental not only for safeguarding the Occupational Health and Safety (OHS) of workers but also for ensuring sustainability of mining companies by enhancing productivity of mine workers^1^. Uncomfortable working conditions can lead to accidents, injuries, and Musculoskeletal Disorders (MSDs). These issues can disrupt operations, incur expenses, and jeopardize the sustainability of mining enterprises. MSDs can result in significant healthcare costs for medical treatment, rehabilitation, and worker compensation. Addressing MSDs benefits workers by reducing pain and disability, thereby decreasing the risk of errors and accidents. Moreover, mining companies can reduce costs associated with MSDs, enhance staff productivity, and contribute to the long-term sustainability and profitability of the mining industry.

Nevertheless, the prevalence of MSDs continues to be a concern with the adoption of mechanization due to the emergence of new ergonomic risk factors such as awkward postures^2–4^, human vibration^5,6^, sedentary postures^7,8^, endurance^9^, workload, and shift work^10^. According to Kumar^11^ and Mayton et al.^12^, mining operators who engage in prolonged periods of operating Heavy Earth-Moving Machines (HEMM) in a stationary posture are at an increased risk of developing MSDs. Despite the rapid mechanization and the incorporation of effective practises and solutions, HEMM operators continue to face the presence of ergonomic risk factors^13,14^. A recent analysis of Global Burden of Disease, 2019 data showed that approximately 1.71 billion people globally live with MSDs, including lower back pain, neck pain, fractures, osteoarthritis, and rheumatoid arthritis^15^. MSDs are at the top of the list in terms of global Years Lived with Disability (YLD) with a surge of 66% from 1990 to 2017^16^. In 2019, 13.78% of all YLD in India were associated with MSDs; this percentage was 11.55% in 1990 which represents an increase of 2.23% in YLD^17^ highlighting the prevalence of MSDs in India.

Ergonomics is a science that emphasizes design and prioritizes the needs of humans within a work system to mitigate the effects of MSDs. Its objective is to improve both human performance and the efficient operation of work system elements^18^. A work system may be conceptualised as an entity in which a person assumes the role of a worker, carrying out a designated operational activity or function within a particular environment^19,20^. According to Boudreau-Trudel et al.^21^, the procedures involved in enhancing the ergonomics and safety of HEMM are inherently complex. The purchase procedures of mining companies^22^ as well as the equipment design processes^22,23^ exhibit some shortcomings. Boudreau-Trudel et al.^21^ demonstrated that using load-haul-dump trucks equipped with enclosed cabins and air conditioning effectively mitigates the risks associated with dust inhalation and falling rock hazards, thereby ensuring the safety and well-being of operators. However, the introduction of these new cabins has introduced additional hazards, such as operators striking their heads against the roof of the cabin or experiencing bodily collisions with various components inside the vehicle. Consequently, this has led to a significant increase in injury rates^21^.

The evaluation of task organisation and workflow within a given context is of considerable importance when assessing different facets of work processes. The main objective of work process assessment is to discover potential risks, inefficiencies, and hazards in a work system. To optimize work process efficiency, it is important to address various factors such as task management, levels of physical exertion, material handling practices, and required mental effort. Researchers emphasize the importance of incorporating these factors into work process design to reduce the likelihood of accidents and enhance overall performance^24,25^. Another study carried out by Mirdad^26^, opined to investigate the effects of process redesign on the reduction of strain experienced in lifting occupations. The results indicated that the implementation of these redesign initiatives not only resulted in an improvement of worker’s safety but also led to an enhancement in productivity. In a similar vein, Zara et al.^27^ examined the impact of process redesign on mitigating strain in lifting jobs, eventually resulting in enhanced worker safety and heightened productivity.

The assessment of ergonomic work tool is of utmost importance in evaluating its design, use, and safety across diverse work settings. The objective of this assessment is to detect any ergonomic deficiencies that may be associated with injuries, pain, and reduced productivity w.r.t work tools. Considerable investigation has been undertaken about the stimulus of ergonomic tool design on the performance and well-being of employees. According to Haruetai and Worachok^28^, the use of hand tools with ergonomic designs was shown to significantly decrease the amount of grip force exerted. Consequently, reducing the likelihood of developing hand-arm vibration syndrome. In a similar vein, Carson^29^ and Kelley^30^ emphasised the need for ergonomic tool analysis to enhance worker comfort and address the issue of cumulative trauma disorders.

Another important element in ergonomic hazard assessment is work posture analysis. The equipment operators employed in mining operations are required to perform their tasks in a challenging posture as a result of the cramped layout of the HEMM cabin. This is due to the lack of abundant space available in underground coalfields and becoming a contributing factor to diminish the worker productivity, as well as the development of MSDs and fatigue among the mine workers^31^. The occurrence rate of MSDs symptoms is notably seen in the lumbar region, torso, cervical area, upper extremities, and knee joints^32^. The prevalent MSDs seen in industrial settings include strains, lower back pain, carpal tunnel syndrome, hernias, and other related conditions^33^. These injuries are mostly attributed to the presence of ergonomic hazards. Various researchers have reached the consensus that there exists a significant correlation between uncomfortable body positions and MSDs^34–39^. The same has been confirmed by the National Institute of Occupational Safety and Health (NIOSH, USA)^40^.

Based on the extensive literature conducted, it clearly indicates that the ergonomic risk assessment involves subjective factor analysis, such as evaluating work processes and tools. An integrated system is necessary to analyse ergonomic risks, including work processes, tools, and postures, and their impact on MSDs among the mine workers. Several studies have highlighted the lack of rapid, robust, and integrated systems for conducting ergonomic risk assessments^41–43^. Moreover, the ergonomic risk assessment requires handling uncertain or imprecise information, where data may be ambiguous or unclear. A robust model is necessary to help the industry avoid the challenges of relying only on individuals’ subjective inputs while performing ergonomic assessments.

Currently, there is a growing trend in the use of fuzzy logic techniques for addressing practical problems and facilitating the analysis of vague data^44^. These strategies have shown their efficacy as a valuable instrument for addressing the intricacies, deficiencies, and ambiguities encountered in many domains. The fuzzy rule-based system (FRBS) is a reliable mathematical method for transforming data into a set of fuzzy rules for the purpose of analysing the complexities inherent in real-world scenarios^45–47^. However, this particular methodology has not been used so far in the assessment of MSDs resulting from ergonomic risks, particularly within the Indian mining sector. Consequently, the Fuzzy Musculoskeletal-disorder Index (FMI) was developed by integrating assessments of work process, work tool, and work posture. This research primarily encompasses of two objectives.

i. To perform ergonomic hazard analysis on HEMM operators in Indian underground mines. This objective is conducted in accordance with the guidelines outlined in the 11th Indian Safety Conference on Mine Safety and the circular released by the Directorate General of Mines Safety (DGMS): DGMS (TECH) circular (MAMID)/08 dt: 29/04/2020^48^.
ii. To proactively appraise the MSDs resulting from ergonomic hazards using the FMI.

## Methodology

The theoretical approach of the study is grounded on the principles of participatory ergonomics (PE) and design science. PE is seen as a strategy within the field of ergonomics studies. It involves a systematic process in which all relevant stakeholders are actively engaged in collaborative problem-solving efforts^49^. Design science and ergonomics have a strong relationship since both disciplines are focused on enhancing human well-being via the application of their respective principles. Design science encompasses a technology-oriented approach, aiming to generate information that can be effectively used for various design and management objectives^50^. Though design science in this study is used only for selection of work tool sub-criteria with the help of extensive literature review. The research did not include the collection of any sensitive personal data from the individuals, and all participants provided voluntary consent to participate. The subjectivity of the data and interpretation was evident in this investigation. In accordance with the principles of design science research, this study did not primarily aim to investigate pre-existing models. Rather, the study sought to contribute new information in the field of design science via a comprehensive analysis of relevant literature, experts’ opinion, and participatory evaluation sessions.

This research complied with the American Psychological Association Code of Ethics and informed consent was obtained from each participant. Approval of all ethical, experimental procedures and protocols was granted by the research and development cell of Indian Institute of Technology (Indian School of Mines), Dhanbad, India. The authors confirm that all the methods were performed in accordance with the guidelines and regulations approved by the research and development cell of Indian Institute of Technology (Indian School of Mines), Dhanbad.

### Participatory ergonomics study framework

There were seven underground mines selected for this study which are located in the eastern region of India employing around 350 HEMM operators. The PE sessions were conducted separately for reach respectively mines at their workplace. The research method included six distinct kinds of activities, denoted as activity I–VI. The first step was the formation of a panel of experts (activity I) tasked with designing a questionnaire to assess ergonomic risk factors. The detailed methodology will be elaborated upon in the subsequent section “Identification of ergonomic risk factors”. Following the creation of the questionnaire, a colloquial conversation was placed at the mine site with the OHS manager (activity II) to ascertain the requirements and potential constraints of the PE procedure. Following the talk, activity III ensued, which includes engaging in a dialogue with the randomly selected 81 HEMM operators. The purpose of this interaction was to elucidate the fundamental aspects of the research and foster their comprehension of the collaborative efforts necessary to effectively execute the study. The sample of 81 HEMM operators consisted of individuals proficient in operating various equipment, such as mine trucks, load haul dumpers, passenger carriers, and multi-utility vehicles. The vehicles under observation were representative of mining vehicles commonly available for purchase on a worldwide scale. During the study, the researchers closely monitored the operators during their regular work shifts, with a primary focus on collecting data on the various duties being performed by the operators. Additionally, the researchers aimed to detect any safety and ergonomics issues that were prevalent in the operators' work environment. In conjunction with on-site direct observations, researchers captured photographs of HEMM operators engaged in their tasks, with the intention of conducting subsequent reviews.

**Figure 1:**
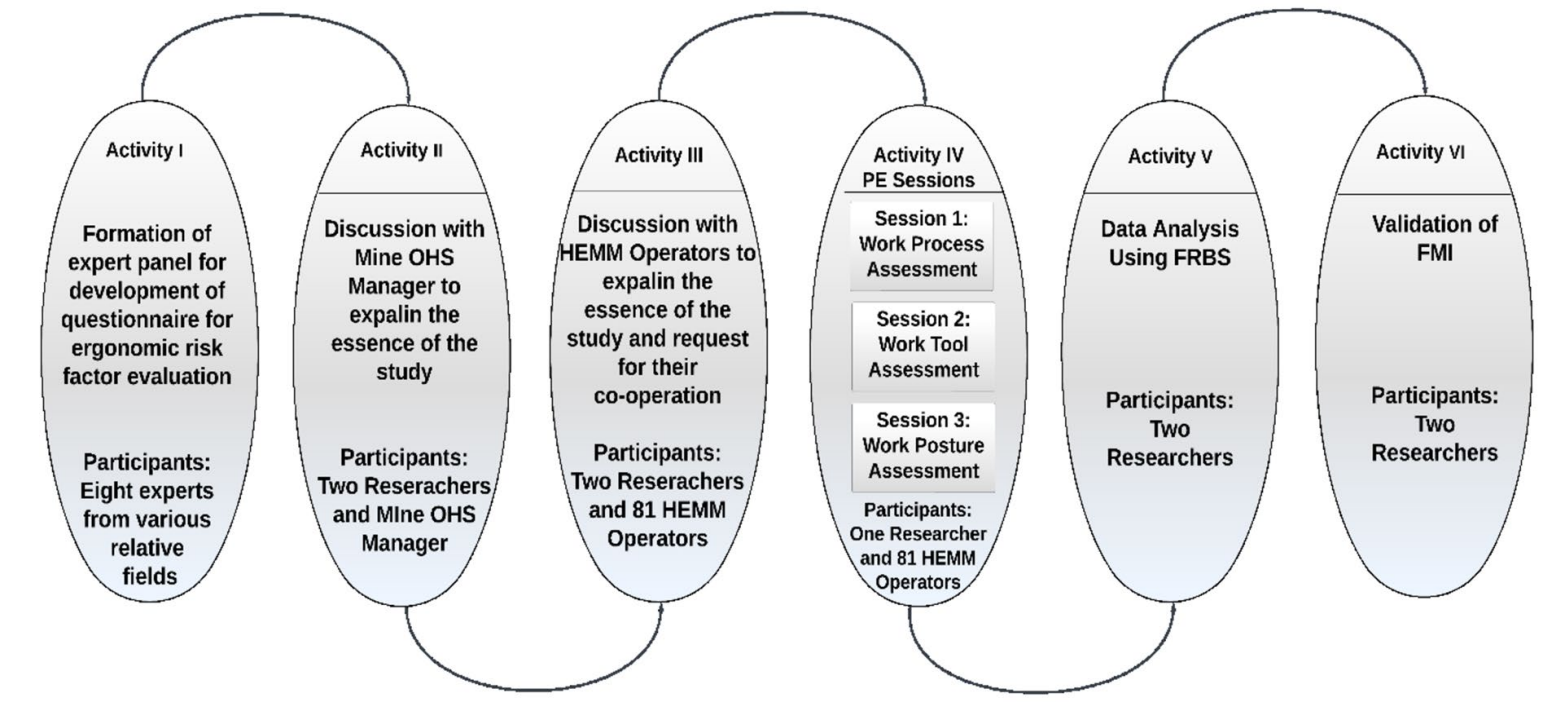
The thematic research process: activities and explanations.

PE sessions (activity IV) start after activity III. A total of three sessions were scheduled (refer to Fig. 1). The format of the PE sessions bore a resemblance to focus group sessions^51^, as they exhibit a semi-structured framework and were assigned for a specific objective to be addressed during each session. The PE sessions were conducted inside the designated meeting rooms located within the mining premises and the workplace. The first PE session (consists of one researcher and 81 operators) was conducted with the objective of administering a questionnaire that focuses on identifying the various hazards connected with the work processes of HEMM operators. During the second PE session, a group of participants consisting of one researcher and 81 operators were asked to assess the risk factors associated with work instruments and assistance by completing a questionnaire. Following the first two PE sessions, the subsequent and final PE session included a total of 81 operators and one researcher. The objective of this session was to assess the presence of any instances of uncomfortable posture shown by the HEMM operators over the course of their jobs. The use of pre-existing photographic material captured during the operators' jobs was employed to facilitate and foster discourse. Subsequently, following the PE sessions, the reliability of the constructed questionnaire was assessed and examined with the Fuzzy Rule Based System (FRBS) (activity V). In the sixth activity, the validation of the FMI was conducted using a two-sample *t*-test.

### Identification of ergonomic risk factors

Following an extensive review of existing literature and the facilitation of a focus-group discussion with experts, a comprehensive set of sub-criteria has been identified for the purpose of evaluating the OHS hazards associated with work processes, work tools, and work postures. According to Drew et al.^52^, scholarly literature suggests that a focus-group discussion often necessitates the participation of 6 to 10 experts. This study included the implementation of a focused group discussion, which included a panel of eight individuals who had expertise in the fields of OHS, mining, environment, and mining industry. The selection of these eight experts was conducted by using purposive sampling approach. The criterion for expert participation included both relevant experiences and topic expertise. A comprehensive set of sub-norms for the questionnaire was developed by drawing upon relevant literature. Subsequently, the chosen questionnaire was presented to a panel of specialists for their evaluation and endorsement. In order to safeguard confidentiality, aliases have been referred instead of the real names of the professionals. The profiles of the specialists are shown in Table 1. Following an extensive review of the existing literature and engaging in consultations with subject matter experts, a complete set of 16 risk factors were identified and then classified. The selection of these characteristics was based on their pertinence to the mining industry, with a specific focus on HEMM operators.

**Table 1:** Expert group member for finalising questionnaire.

Name	Job title	Organization/company	Experience	Degree	Field
Expert 1	Faculty	IIT (ISM), Dhanbad	20	PhD	Mine Safety and Ergonomics
Expert 2	Faculty	IIT (ISM), Dhanbad	18	PhD	Environment Engineering
Expert 3	Faculty	IIT, Kharagpur	18	PhD	Industrial Engineering
Expert 4	Faculty	IIT (ISM), Dhanbad	35	PhD	OHS (Mining)
Expert 5	Manager	Study Area Mines	17	B. Tech	Mine
Expert 6	Manager	Study Area Mines	22	B. Tech	Mine
Expert 7	Consultant	Private Firm	7	M. Tech	Ergonomist
Expert 8	Consultant	Private Firm	10	M. Tech	OHS

Furthermore, the assessment of a questionnaire survey's quality may be conducted by evaluating its validity and reliability. Validity pertains to the extent to which a measurement accurately captures the intended construct or phenomenon. It concerns whether the measurement instrument is really assessing what the researcher intends it to assess. According to Bolarinwa^53^, two significant types of validity have importance in research: content validity and construct validity. The establishment of content validity for a measuring instrument included a meticulous examination of the domain that the questions represented. The determination of this criteria is subjective in nature and is most accurately assessed via the evaluation of expert perspectives and analysis of relevant scholarly works, as elucidated in the preceding paragraph. The establishment of construct validity for a measuring instrument may be achieved by doing a statistical study of the measurements of questionnaire data using factor analysis. The first step was doing a factor analysis to examine the factor loadings of each item with respect to the variables (factors) of interest. According to Rahman^54^, a factor loading of 0.3 or more was deemed to be statistically significant. Ultimately, the items that met the criteria for factor loading were valid indicators of the construct. The evaluation of data reliability derived from a questionnaire survey is determined by assessing the internal consistency of questions aimed at measuring the same concept. Cronbach's alpha is a commonly used metric for assessing internal consistency. The consistency of a collection of questionnaires is quantified by a metric. The measurement of internal uniformity reliability for each of the manifest variables was conducted using Cronbach's alpha. A satisfactory alpha value is often considered to be 0.7 or more, however in some cases, such as exploratory studies, a value of 0.6 may be deemed acceptable^55^.

### Classification of the risk factors

The risk features identified were classified into three categories: (i) work process, (ii) work tool, and (iii) work posture. Eight specialists from various fields (Table 1) in India were contacted for this purpose. Out of 16, eight questions were selected for the assessment of work process (Table 2) and eight questions for the assessment of the work tool assessment (Table 3) from literature and expert’s opinion. Moreover, the factor loading for construct validity and Cronbach’s alpha for the reliability of the selected questionnaire were shown in Tables 2 and 3 for work process and work tool analysis, respectively. Rapid Upper Limb Assessment (RULA) performs the work posture assessment. The RULA ergonomic assessment method takes into account of biomechanical and postural load needs associated with occupational duties and demands on the neck, trunk, and upper extremities^56^. The assessment of necessary body position, force, and repetition is conducted using a one-page worksheet. The assessment involves recording scores for several body regions. Specifically, the arm and wrist scores are recorded in part A, while the neck and trunk are evaluated in part B. Once the data pertaining to each workplace location has been gathered and evaluated, tables within the form are used to aggregate the variables associated with risk factors. This process culminates in the creation of a singular score, which serves as an indicator of the amount of risk for MSDs as described in the following manner:

- A grand score = 1–2 indicates that posture is negligible risk.
- A grand score = 3–4 indicates that posture is low-risk, and changes may be needed.
- A grand score = 5–6 indicates that posture is high-risk level, and further investigation is needed to change posture as soon as possible.
- A grand score = 7 indicates that posture is very high-risk level, and immediate change in the posture is needed.

**Table 2:** Developed questionnaire, construct validity and reliability of work process assessment.

Sl. no.	Question	Factor loading	Cronbach’s alpha	References
1	How is the unevenness of the haul road for HEMM operation?	0.682	0.831	^58–61^
2	Are the turnings causing you to take an awkward position?	0.816		
3	How is the temperature in the workplace?	0.823		
4	How is the sound in the workplace?	0.878		
5	How is the lighting in the workplace?	0.813		
6	How frequently is the HEMM moved to perform the task in your workplace?	0.311		
7	How is the workload in the shift?	0.451		
8	Is there any provision for job rotation in your workplace?	0.638

**Table 3:** Developed questionnaire, construct validity and reliability of work tool assessment.

Sl. no.	Question	Factor loading	Cronbach’s alpha	References
1	How much exertion or force is to be used to operate HEMM?	0.687	0.866	^58,59,62,63^
2	Are tools causing forceful pinch grips?	0.805		
3	Is the HEMM vibration causing MSDs pain?	0.888		
4	How much hard is a lever for operating HEMM?	0.506		
5	How is the design of the seat w.r.t posture adoption?	0.716		
6	How frequently do you use too many tools/levers simultaneously?	0.835		
7	Are HEMMs equipped with handles to enter/exit that are suitable for most workers?	0.615		
8	Is there a preventive maintenance program to keep HEMM operating as designed?	0.689		

### Analysis of work posture of the HEMM operators

If the worker is operating in an awkward posture over a long stretch of time period, the worker may develop stress spots in different parts of anatomy of the body, eventually deteriorating their health^57^. The operator's degree of comfort during operation is influenced by his/her body position. Evaluation of posture entails in finding improper postures adopted by operators when operating a machine.

Various biomechanical analyses were available to evaluate the effect of awkward posture resulting in MSDs. Fatigue failure theory investigates how repeated loading and stress cause structural deterioration and failure in biological tissues, especially joints^58^. 3D Static Strength Prediction Program, developed by the University of Michigan, is a software program that forecasts the static strength required for different manual actions such as lifts, presses, pushes, and pulls^59^. According to Gallagher's study, MSDs might be the outcome of a fatigue failure process. This unified paradigm seeks to explain why physical risk factors contribute to the development of workplace MSDs. Chronic or recurrent stress degrades tissues over time, resulting in discomfort, inflammation, and malfunction^60^. Moreover, there were several observational methods available for evaluating the posture adopted by mine workers namely Ovako Work Analysis System (OWAS), Occupational Repetitive Actions (OCRA), Rapid Entire Body System (REBA), and Rapid Upper Limb Assessment (RULA) etc. These observation methods were relatively simple, easy to implement in real-world settings, and cost-effective.

The study focuses on HEMM operators who consistently work in a seated posture, with their legs supported by the floor. Hence, the assessment of the upper limb is necessary and may be effectively conducted utilizing the RULA approach. Therefore, this study used the RULA methodology, which primarily focuses on evaluating the upper extremities. Moreover, RULA is cost-effective, simple and very less time-consuming technique. Ergo Master software (NexGen ergonomics) was used to analyse posture with the aid of RULA. Videos and photographs were used in this research to examine the worker's motions and stances throughout various duty cycles.

The Ergo Master programme allows the importation of digital photos from a video recording while the operators were working. The programme's catalogue will enable us to save and salvage the stance score based on the input photographs (NexGen ergonomics). The scores above 2 is considered as an awkward posture. As the score increases, the risk of MSDs increases.

### Development of fuzzy musculoskeletal-disorders index (FMI)

The foundation of FMI is rooted on the theoretical framework of fuzzy set theory and fuzzy logic. The development of fuzzy set theory was motivated by the need of effective model systems, operating in situations characterised by the presence of uncertainty and imprecision. This research incorporates a technique known as FMI, which aims to tackle the issue of accurately mapping input and output variables that possess vague or imprecise descriptions^64^. In the domain, two often used techniques for fuzzy inference include the Mamdani fuzzy model and the TSK (Takagi Sugeno Kang) fuzzy model. The Mamdani fuzzy inference model employs rules that are developed from historical data and past experiences^65^. On the other hand, the TSK fuzzy model depends on rules formed from a particular collection of input–output data^66^. This study favours the Mamdanis inference system for its inherent characteristics, compatibility with human inputs, improved interpretability, and dependence on rule-based procedures.

**Figure 2:**
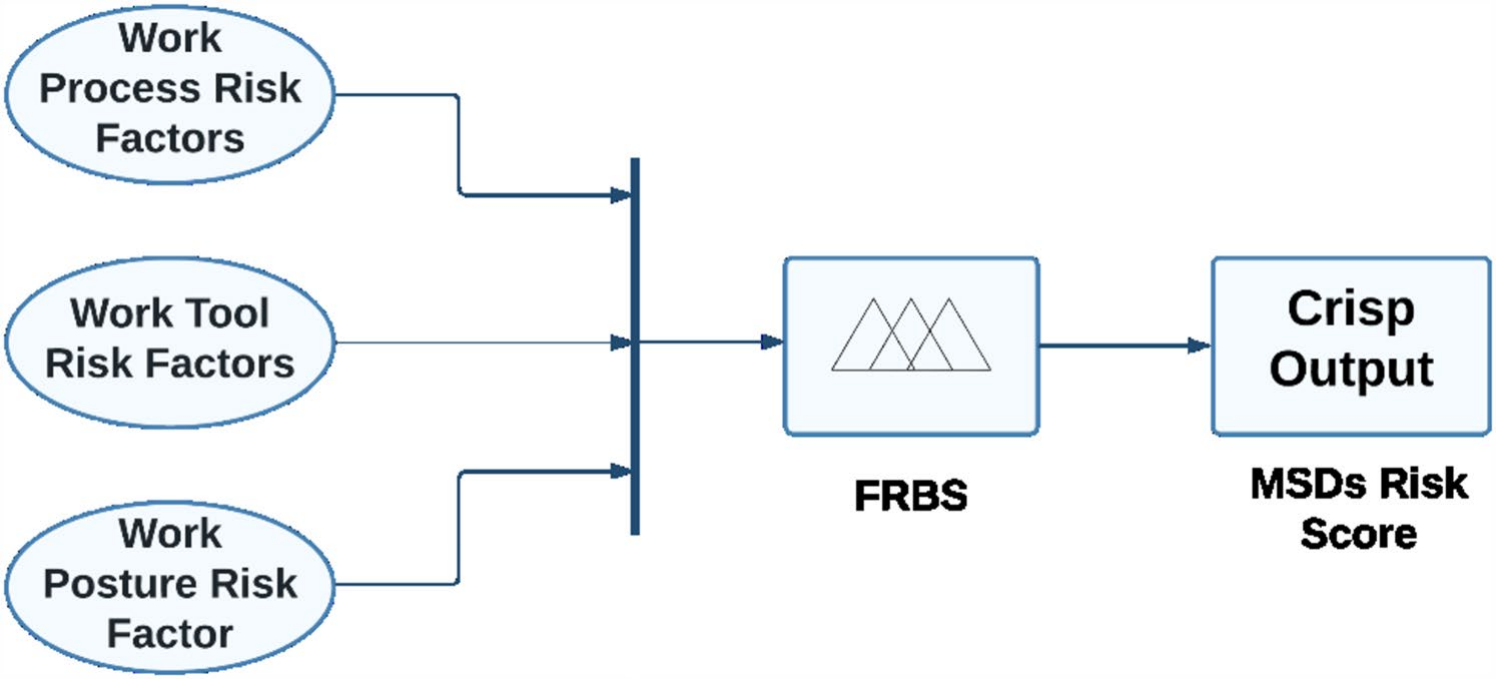
Proposed FMI model.

Furthermore, it has reaped substantial acknowledgement among the academic community^67–69^. In additional, it has been noted that Mamdani’s FRBS have the capability to be developed using the assessments provided by a limited group of specialists^70^. Hence, the Mamdani fuzzy model has been used in this study. The model developed for assessing the risk of MSDs is shown in Fig. 2. The development of the proposed FMI concept encompasses four primary stages. These stages are delineated below.

(a) Selection of input and output variables: The initial phase in developing an FMI model involves identifying and selecting input variables. The inclusion of input variables has the potential to increase the complexity of the model. On the other hand, the absence of input variables may impact the robustness of the FMI model. Hence, the present study emphasises three distinct criteria for selecting input variables, namely work process risk factors, work tool risk factors, and work posture. The dependent variable is operationally defined as the risk score for MSDs. The process of selecting input and output variables also entails using logical reasoning, which is operationalized via categorising each variable. For example, the work process may be classified into five categories: very low, low, medium, high, and high. Similarly, the work tool can be categorised as very low, low, medium, high, and very high. The work posture can be classified as low, medium, high, and very high. Lastly, the level of risk can be categorised as very low, low, medium, high, and very high.

(b) Formulation of membership functions: The linguistic designations for each variable are articulated via the use of sets. The fuzzy set is often defined by its membership functions. Many categories of membership functions are applicable to an FMI model. Gaussian and triangular membership functions have shown superior performance compared to other forms of membership functions and are often used in fuzzy rule-based models. Zhao and Bose^71^ conducted a comparative analysis of many kinds of membership functions and arrived at the conclusion that the triangular membership function exhibits greater performance when compared to other membership functions. The implementation of triangular membership functions is straightforward and has minimal computational complexity. The use of triangular membership functions has been employed in the current investigation due to the aforementioned rationales. The triangle membership function is characterised by three parameters: [a, b, c]. Mathematically, the phenomenon may be stated in the following manner:

1 $$ {\text{triangle}}\; \left( {x;\;a,\;b,\;c} \right) = \left\{ {\begin{array}{*{20}c} {0,} & {\quad x \le a} \\ {\frac{x - a}{{b - a}},} & {\quad a \le x \le b} \\ {\frac{c - x}{{c - b}},} & {\quad b \le x \le c} \\ {0,} & {\quad c \le x} \\ \end{array} } \right\} $$

The linguistic value is determined by the input parameters a, b, and c, while the range of the input parameters is denoted by x.

(c). Formulation of IF–THEN rules: After determining the membership functions for the input and output variables, inference rules may be formulated based on existing experience and knowledge. The proposed model consists of three input variables and one output variable, each having three membership values. The model conforms to the Mamdani rule framework, which is characterised by its adherence to IF logic. As a result, the suggested FMI is comprised of a total of 100 rules (5 × 5 × 4).

(d). Interface and Defuzzification: The Mamdani fuzzy inference method was ultimately chosen to aggregate the suitable output and perform defuzzification, resulting in the acquisition of a crisp output. Defuzzification refers to the process of converting fuzzy output, which is characterised by imprecise or uncertain values, into crisp output, which consists of precise and well-defined values. In this section, the computational complexity of the FMI model is particularly evident. This stage provides a quantitative result. Various defuzzification strategies may be used in FMI models, such as the bisector of area, centre of area, mean of maximum, largest of maximum, and smallest of maximum approaches^72^. The centroid approach was used in this research for the purpose of defuzzification.

2 $$ MSDs \;Risk\; Score = \smallint x\mu_{{\text{out }}} (x)\;dx/\smallint \mu_{{{\text{out}} }} (x)\;dx $$

**Figure 3:**
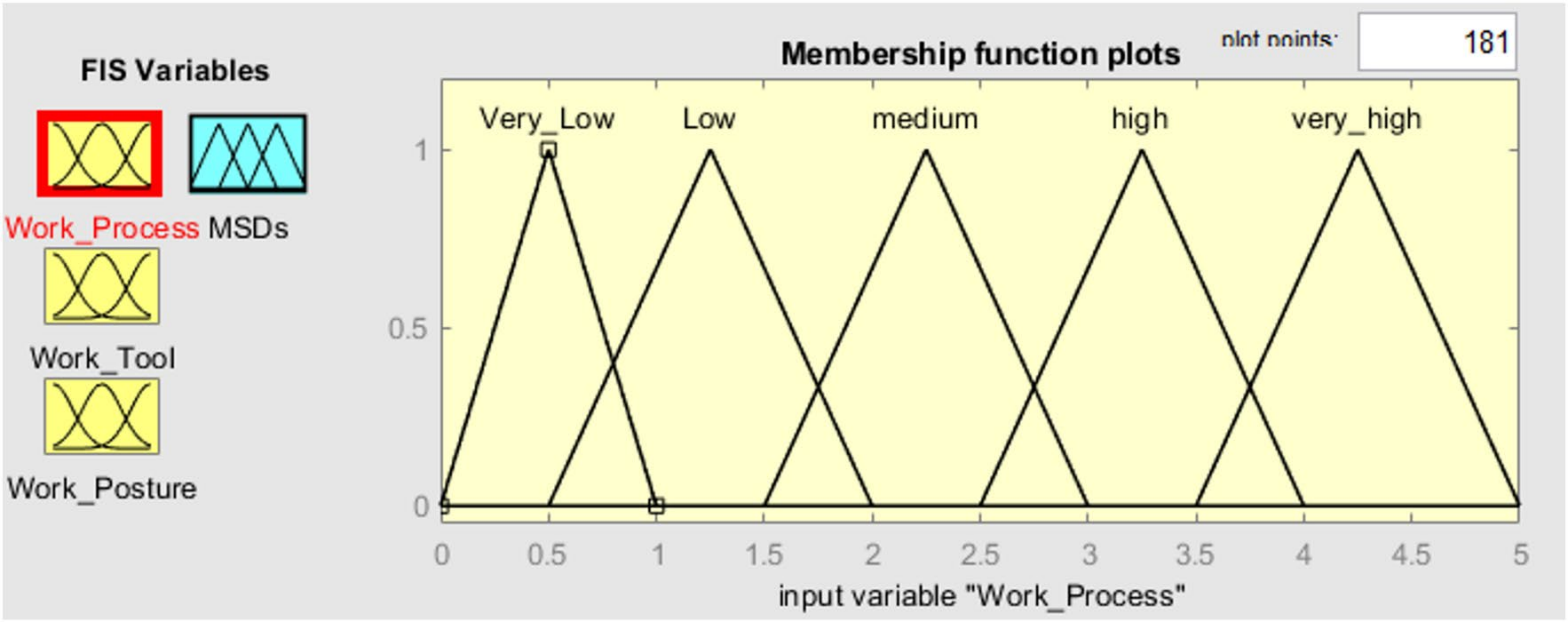
Membership functions plot for work process factor.

**Figure 4:**
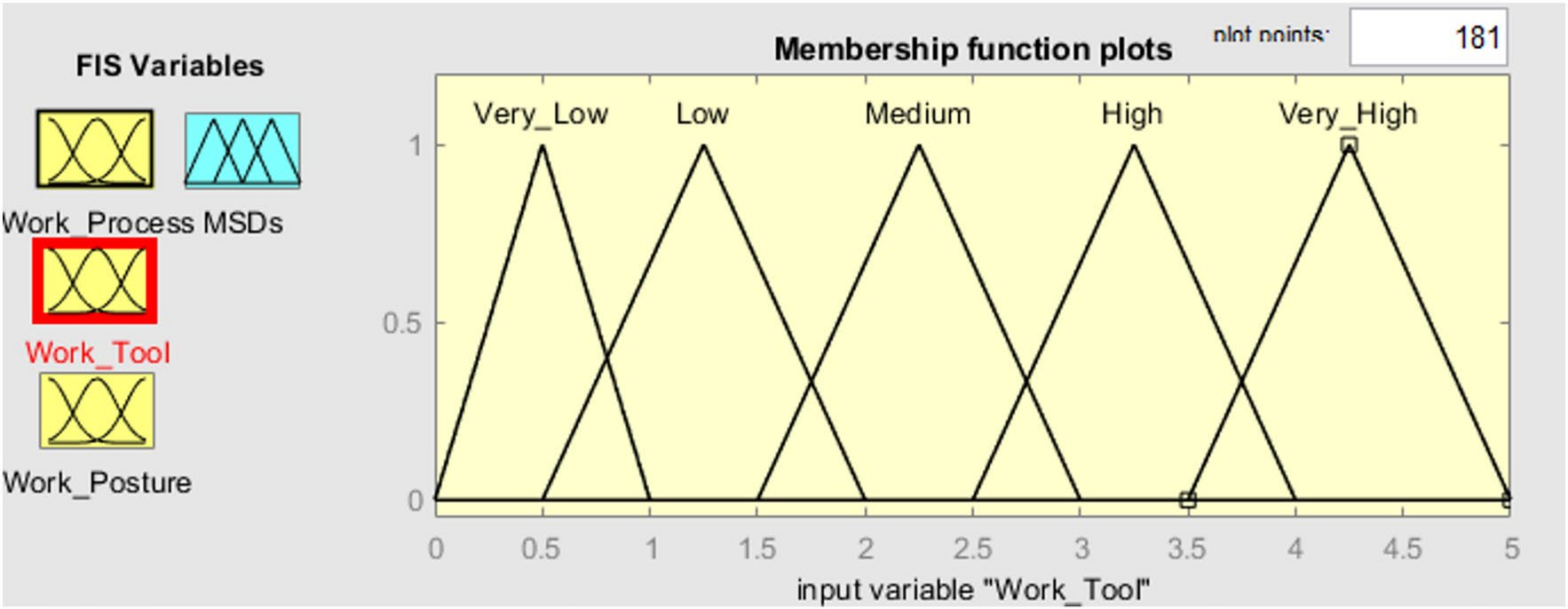
Membership functions plot for work tool factor.

**Figure 5:**
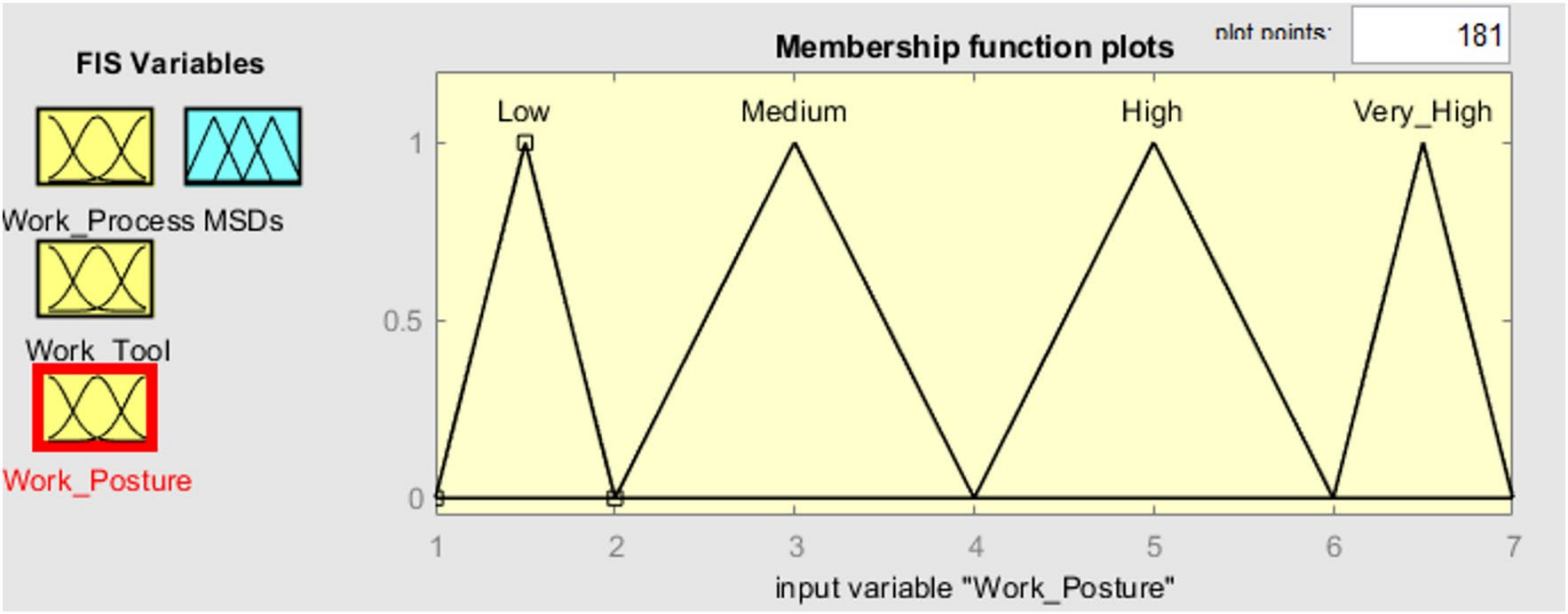
Membership functions plot for work posture factor.

**Figure 6:**
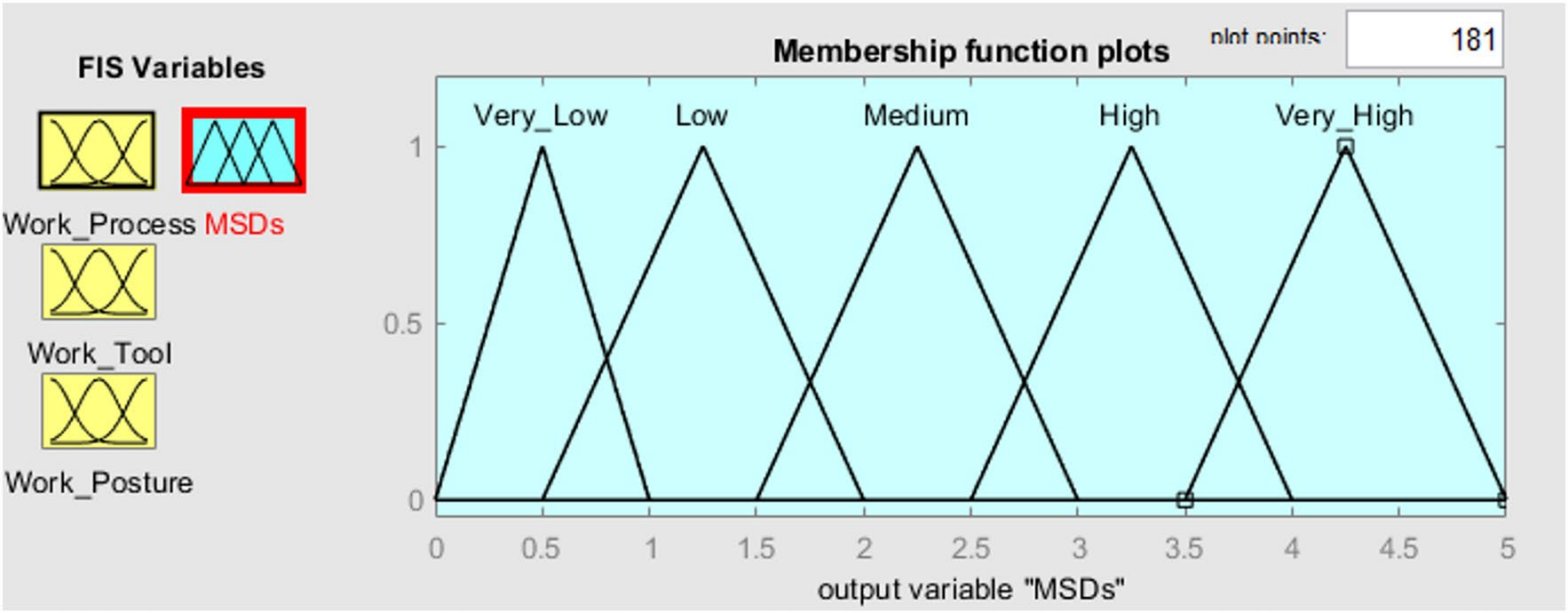
Membership functions plot for MSDs risk.

**Figure 7:**
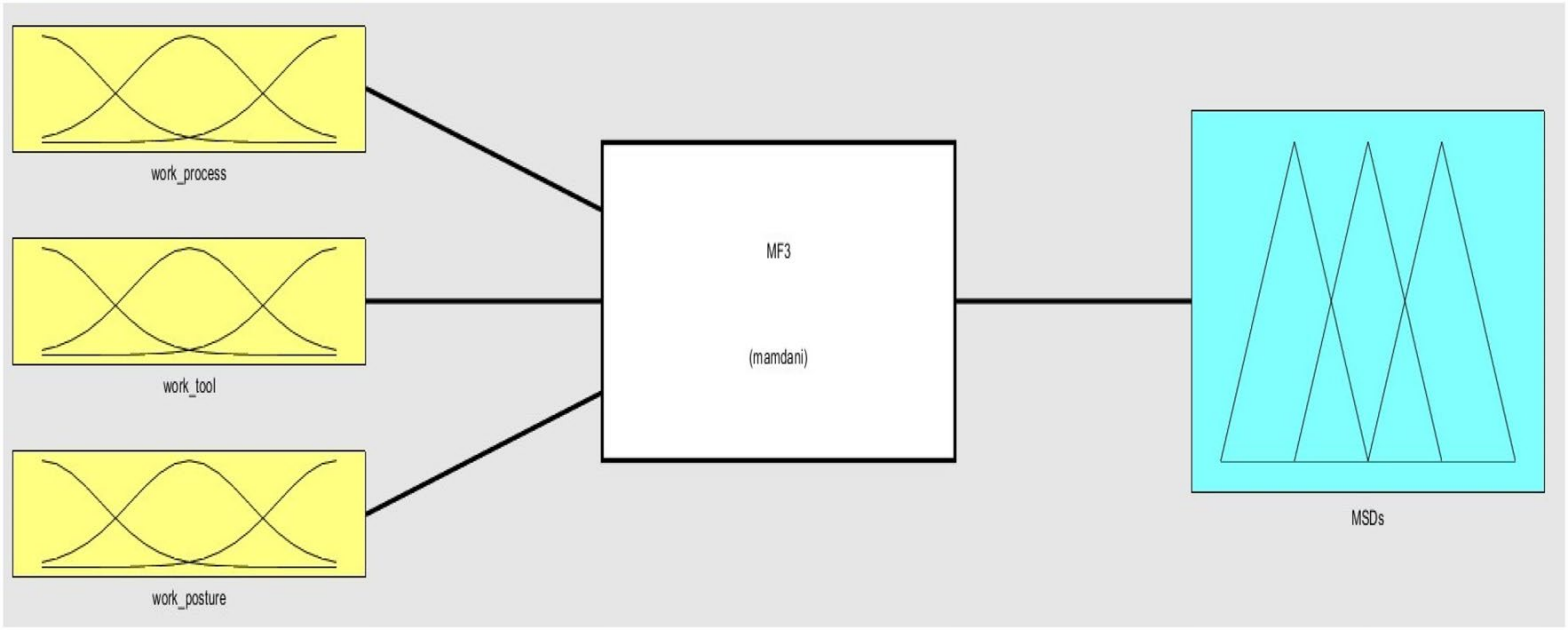
FMI of MSDs risk evaluation.

To verify the established FMI model, seven subterranean mines situated in the eastern region of India were chosen as the subjects for evaluating ergonomic hazards. Three inputs and one output have been chosen in the FMI based risk assessment model for the determination of MSDs of HEMM operators in seven underground mines. The three inputs consist of the assessment criteria that have been previously chosen, namely the risk factors associated with work processes, work tools, and work postures. The resultant outcome of the FMI model pertains to the ultimate level of MSDs risk. The questionnaires used to evaluate work process risk factors and work tool risk factors had a Cronbach alpha coefficient of over 0.7, indicating that the questionnaires utilised were trustworthy and free from bias. As several membership functions like triangular function, trapezoidal function etc. had been widely used in developing fuzzy membership functions. The expert’s opinion was considered to decide the type of fuzzy membership function for each criterion. Moreover, even the linguistic characteristics values were decided based on the expert’s opinion as there is no such specific rules to decide the linguistic characteristics values. Figure 3 depicts the plot of the fuzzy membership function used for the evaluation of work processes. The membership functions assigned to the work process assessment criteria are as follows: very low [0 0.5 1] (triangular fuzzy number), low [0.5 1.25 2] (triangular fuzzy number), medium [1.5 2.25 3] (triangular fuzzy number), high [2.5 3.25 4] (triangular fuzzy number), and very high [3.5 4.25 5] (triangular fuzzy number). The membership functions presented above serve to show the various risk variables associated with the work process within the designated research region. Figure 4 displays the plot of the fuzzy membership function for the evaluation criteria of work tools. In this particular instance, the five membership functions are defined as follows: very low [0 0.5 1] (triangle fuzzy number), low [0.5 1.25 2] (triangular fuzzy number), medium [1.5 2.25 3] (triangular fuzzy number), high [2.5 3.25 4] (triangular fuzzy number), and very high [3.5 4.25 5] (triangular fuzzy number). Figure 5 depicts the plot of the fuzzy membership function for the criteria associated with work posture. The definitions of the four membership functions are as follows: the low membership function is represented by the triangle fuzzy number [1 1.5 2], the medium membership function is represented by the triangular fuzzy number [2 3 4], the high membership function is represented by the triangular fuzzy number [4 5 6], and the very high membership function is represented by the triangular fuzzy number [6 6.5 7]. The membership functions pertaining to the output variable of MSDs risk were eventually developed. The membership functions for the output variable are shown in Fig. 6. In this particular circumstance, the three membership functions may be delineated as follows: very low [0 0.5 1] (represented by a triangular number), low [0.5 1.25 2] (represented by a triangular number), medium [1.5 2.25 3] (represented by a triangular number), high [2.5 3.25 4] (represented by a triangular member), and very high [3.5 4.25 5] (represented by a triangular member) correspondingly. The FMI model was constructed based on the aforementioned procedure using Fuzzy Logic module in MATLAB 2021a. Figure 7 displays a schematic illustration of the FMI model that has been developed for the purpose of evaluating the risk linked to MSDs.

### Development of MSDs questionnaire

The musculoskeletal symptom questionnaire was developed to estimate the perception of Musculoskeletal Disorders (MSDs) among the general population. The questionnaire was developed using the literature review and experts’ suggestion. Literature review was conducted to collect the various questions that evaluate MSDs perception. Later, the same is kept before the expert’s panel to sort out the questions from the questionnaire developed using literature review that fits to evaluate the MSDs among the mining HEMM operators’ population. The developed questionnaire was put before HEMM operators for their response such that the results of the MSDs questionnaire can be used to validate the results of developed FMI.

## Results of developed FMI

### Postural assessment results

**Figure 8:**
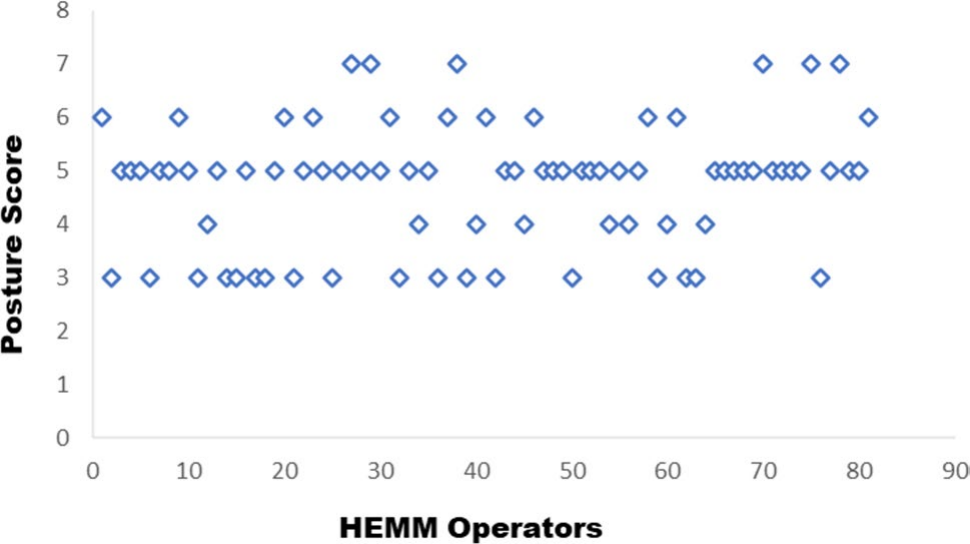
Postural score variation among HEMM operators.

The postural analysis result shows mean score of 4.74, exhibiting a variability between 3 and 7. The mean score of 4.74 states that the posture adopted by the HEMM operators was at high risk and further investigation is needed to change the posture as soon as possible. The results highlight the essence of implementing the minimizing measures to reduce the effect of awkward posture on MSDs. Figure 8 highlights the work postures adopted by the various HEMM operators of all the seven underground mines. Eighteen operators had a posture score of 3, and eight had a score of 4, indicating that the posture adopted was at low risk. Thirty-eight and eleven operators had a posture score of 5 and 6, respectively, indicating that the posture adopted by the operators was at high risk. Six operators had a postural score of 7, indicating that the posture adopted by HEMM operators was at very high risk. Fifty-five operators out of eighty-one (67.9%) found that the postures adopted were at higher risk. This sheds the light on the importance of awkward work postures impact on the MSDs risk of the HEMM operators.

### Results of developed FMI

**Figure 9:**
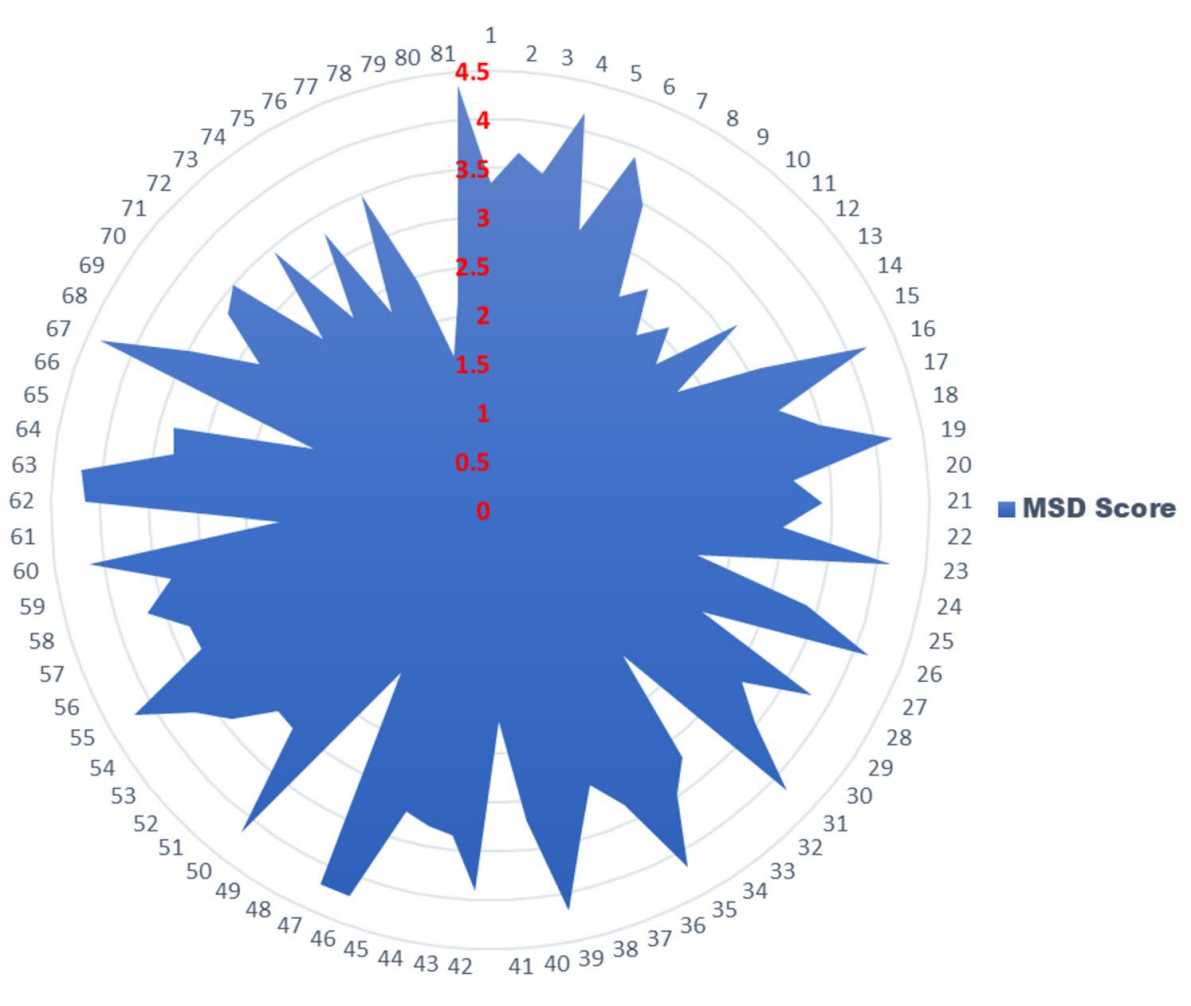
FMI result variation of MSD among HEMM operators.

The FMI results show the average MSDs score is 3.69 (range of 1.62–4.36) which states that the HEMM operators were undergone high to very high risk of MSDs. The variation in the FMI resulted MSDs score of the HEMM operators is shown in the Fig. 9. Three operators were impacted with low to medium MSDs risk, five HEMM operators had the MSDs risk of medium to high and six operators were resulted in high to very high MSDs risk. Twelve HEMM operators experienced medium MSDs risk, thirty-eight operators faced high risk, and seventeen operators impacted with very high MSDs risk.

**Figure 10:**
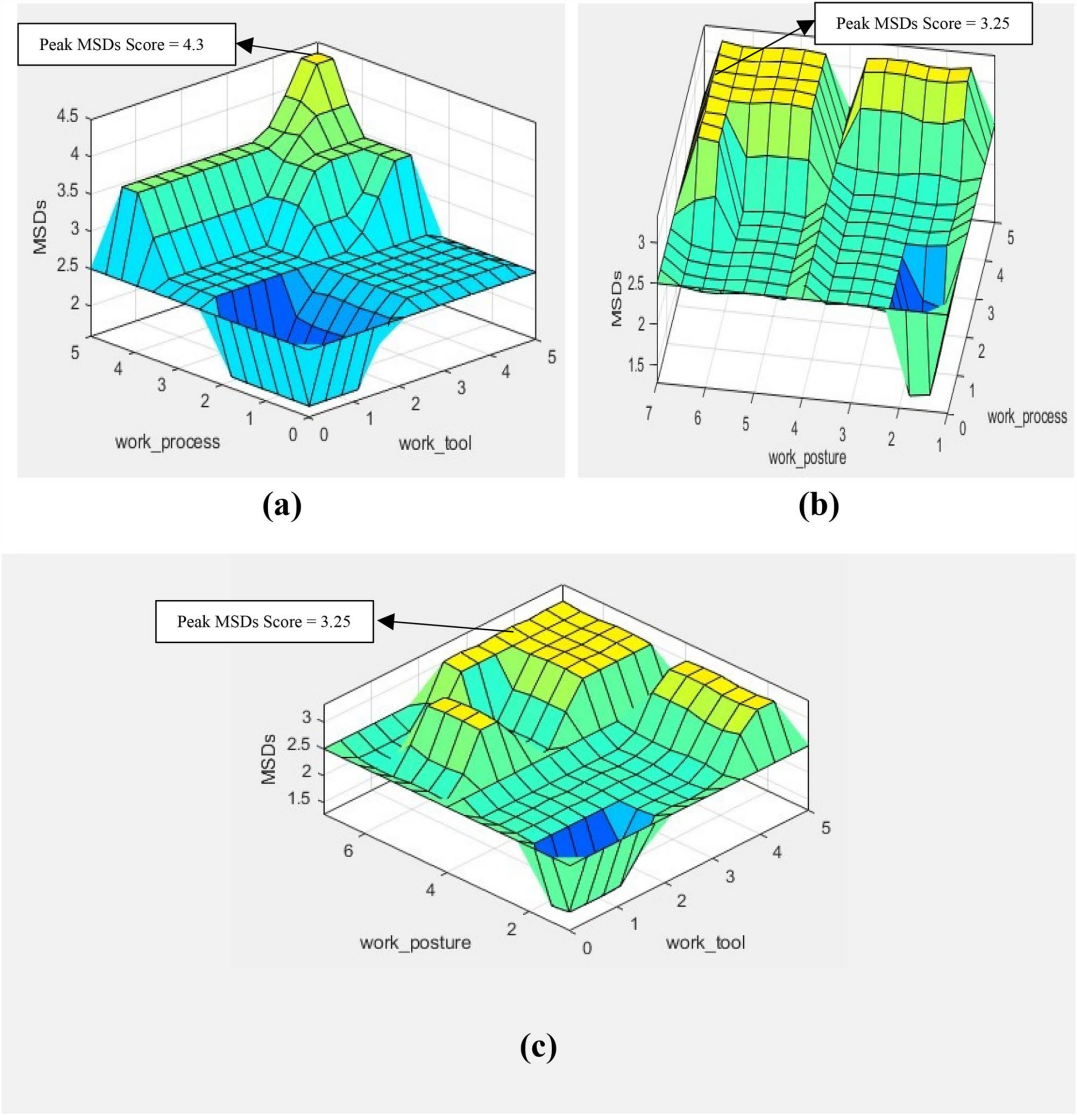
Echelons attained from FMI (**a**) process vs tool (**b**) posture vs process (**c**) posture vs tool.

Figure 10 depicts the interconnections among the input factors and the resultant output, especially demonstrating the risk score for MSDs derived from these inputs. It is evident that the system under consideration consists of three inputs and one output, hence necessitating a cumulative of four dimensions is required. However, the practicality of depicting four dimensions is limited. Surface plots are used as a means to illustrate the relation between two independent variables and a dependent variable. In this scenario, the Z axis indicates the output, whilst the X and Y axes correspond to the inputs. Figure 10a explains the relationship between work process, work tool, and the MSDs risk score. Here, work posture is fixed. Figure 10b explains the relationship between work process, work posture, and the MSDs risk score (work tool is fixed). Whereas Fig. 10c explains the relationship between work tool, work posture, and the MSDs risk score (work process is not included). In all the surface plots, the contour lines indicate regions of MSDs score.

Figure 10a illustrates the significant influence of both work process and work tool inputs on the output MSDs risk score, indicating their sensitivity to this metric. The MSDs score resulted due to influence of both work process and work tool was 4.3. Whereas MSDs scores were 3.25 and 3.25 due to combined influence of work process, work posture (Fig. 10b) and work tool, work posture (Fig. 10c) respectively. This further provide evidence that both the work tool and work process have a greater impact on the MSDs risk in comparison to combined significance of work posture, work process and work posture and work tool.

It is vital to validate the FMI as it is centred on the rules framed. Though the rules were framed based on the literature and suggestions of expert group formed, it is important those fuzzy rules should estimate the MSDs that replicate the actual field MSDs. For achieving this, nested fuzzy models using various combinations of rules were conducted based on expert committee suggestions. Finally, FMI model is finalized based on the comparison with actual output which was discussed in the succeeding section through the validation of FMI.

## Validation of FMI

To validate the FMI output, a MSDs assessment questionnaire (Table 4) was developed. The questionnaire was developed based on previously available literature^73,74^, to evaluate the MSDs of HEMM operators in underground mines. The developed questionnaire was presented to the HEMM operators for their response. The construct validity and reliability of the questionnaire were satisfied. The factor loading, and Cronbach’s alpha for the same is shown in Table 4. The FMI output was validated through hypothesis testing using a two-sample *t*-test in SPSS version 25. A two-sample *t*-test was performed to test the hypothesis whether there is a significant difference between actual MSDs collected through questionnaire in the field and estimated MSDs through FMI. The two-sample *t*-test was conducted in SPSS to test the following hypothesis:

**Table 4:** Developed questionnaire, construct validity and reliability of MSDs risk.

Sl. no.	Question	Factor loading	Cronbach alpha	Reference
1	How much trouble do you have in neck?	0.771	0.729	^73,74^
2	How much trouble do you have in shoulder?	0.653		
3	How much trouble do you have in upper back?	0.256*		
4	How much trouble do you have in elbows?	0.127*		
5	How much trouble do you have in wrist?	0.008*		
6	How much trouble do you have in lower back?	0.543		
7	How much trouble do you have in hips/thighs?	0.441		
8	How much trouble do you have in knees?	0.638		
9	How much trouble do you have in ankles?	0.289*		
10	At the time of initial onset of the trouble, what was your age?	0.149*		
11	Have you been prevented from carrying out normal activities?	0.601		
12	Have you ever been hospitalized because of the trouble or to any physician?	0.47		
13	What was the time span of the discomfort?	0.474		
14	Have you ever had to change the shift because of the trouble?	0.729		
15	How frequent do you take medication because of trouble?	0.759		
16	Have you been given ergonomic training specifically for your job?	0.044*		

*Indicates the discarded items as the factor loading was less than 0.3.

Null hypothesis (H0) = there is no significant difference between the field MSDs Score and Fuzzy MSDs Score.

Alternative hypothesis (H1) = there is a significant difference between the field MSDs Score and Fuzzy MSDs Score.

The *t*-test results (Table 5) show there was no significant difference between field MSDs Score (M = 3.25, SD = 0.688) and Fuzzy MSDs Score (M = 3.39, SD = 0.696); t (79) = − 1.31, p = 0.192. Additionally, the assumption of homogeneity of variances was tested and satisfied via Levene’s *F* test, F (79) = 0.101, p = 0.751.

**Table 5:** Two sample *t*-test results.

Parameters	n	Mean (M)	Standard deviation (SD)	t-score	Df	p-value	Decision
Field MSDs Score	81	3.25	0.688	− 1.31	79	0.192*	Accept H_0_
Fuzzy MSDs Score	81	3.69	0.696				

*P-value is insignificant.

## Discussion

This study focuses on the results and ramifications of a thorough ergonomic analysis conducted on operators of HEMM in Indian underground mines. The analysis especially examines ergonomic risks constructs namely work process, work tool, and work posture. This study also focuses on influence of work process, work tool and work posture on the MSDs. The study emphasizes the use of an FMI as a means of assessing the MSDs caused due to ergonomic hazards.

The study results indicate that ergonomic risk factors continue to be present in the job of HEMM operators in Indian underground mines. The risk factors include several elements such as uncomfortable body positions, exposure to human vibration, prolonged periods of sitting, physical endurance requirements, high workload demands, and working in shifts. Despite concerted attempts to automate operations and apply effective practices, the high incidence of MSDs among mining operators continues to be a significant problem^13,14^. The prevalence of employees being exposed to ergonomic hazards in Indian mines underscores the need for implementing efficacious ergonomic solutions.

As stated earlier, the research is in accordance with the concepts of PE and design science. The research endeavours to effectively tackle the intricate ergonomic and safety issues related to heavy mining equipment by actively engaging stakeholders in the process of problem-solving. Unforeseen dangers have arisen because of deficiencies in buying procedures and equipment design^75^. The results underscore the need of adopting a holistic strategy towards the ergonomic design and safety measures within the mining sector.

The assessment of ergonomic work processes is of utmost importance in the identification of possible hazards, inefficiencies, and dangers. The research highlights the need of integrating ergonomic principles into work process design by examining characteristics such as job repetition, force exertion, physical material handling, and cognitive stress. The study done by Zare et al.^27^ also examines the effects of process redesign on the reduction of musculoskeletal strain during manual lifting activities. The findings of this research provide more evidence in favour of the beneficial outcomes associated with ergonomic interventions. The implementation of such redesign initiatives not only serves to improve the safety of workers, but also has a positive impact on overall productivity levels of the mines.

The evaluation of the attributes of work instruments has significant importance in guaranteeing their design, use, and safety within work settings. The use of hand tools that are intentionally built with ergonomic considerations has shown efficacy in lowering grip force and mitigating the potential for hand-arm vibration syndrome. The value of analysing instruments to enhance worker comfort and avoid trauma disorders has been underscored by several research undertaken by Haruetai and Worachok^28^, Carson^29^, and Kelley^30^. The studies underscore the need of taking into account ergonomic equipment design in order to improve both worker performance and well-being.

Contemporary research presents FMI as a proactive method for evaluating the effect of ergonomic jeopardies on MSDs. The present FMI integrates assessments of work processes, work tools, and work postures to effectively handle the intricate and unpredictable aspects encountered in real-world situations. The use of fuzzy logic methodologies is a helpful instrument for manipulating imprecise data and managing the complexities inherent in ergonomic risk assessments. Nevertheless, it is crucial to acknowledge that using FMI for assessing MSDs caused by ergonomic risks in the mining sector is an innovative methodology that may require more verification and investigation.

Through the study results and the field observations, few of the recommendations are suggested:

i. The management should develop standard hazard management plan for work process design particularly regarding the haul road design (it was observed in the study area that dangerous turnings with uneven abrupt surface), illumination, heat stress, noise etc., and the hazard management plan should effectively implement in the study area to provide comfort to the workers.
ii. The management should consider the ergonomic design in the HEMM procurement and encourage the manufacturer to provide ergonomically designed HEMM. In the case of already procured HEMM ergonomic specialists may be involved in redesigning seat, cabin and levers considering ergonomic principles. Periodic maintenance of the HEMMs should be done considering the human vibration and ease of work tool usage.
iii. It has to be acknowledged that improvement in the work process and work tool design will prevent the operators adopting uncomfortable postures. However, it is also to be acknowledged that the awareness of the operators also plays a vital role in adoption of comfortable posture (use of seat belt, adjusting the seat as per his comfort). Therefore, the management should bring enlightenment among the operators by conducting training and awareness programs.
iv. Regular ergonomic assessments are essential to identify problematic postures, followed by redesigning workstations to promote neutral postures. This includes adjusting the height of work surfaces, providing adjustable chairs, ergonomic tools, and ensuring frequently used items are within easy reach.
v. Implementation of assistive devices such as lifting aids, ergonomic tools, and anti-fatigue mats can significantly reduce physical strain on workers by minimizing the need for awkward postures and repetitive movements.
vi. Encouraging regular breaks and job rotation is another effective strategy to prevent prolonged exposure to ergonomic hazards, thereby reducing fatigue and the risk of developing MSDs.

Moreover, it is crucial to recognize and address the study’s limitations. Firstly, the research was carried out within a narrow geographical area, focusing on a restricted number of underground mines and HEMM operators. Hence, it is important to note that the results of this study may lack generalizability to mining settings or HEMM operators on a global scale. Secondly, the data gathered in the research, including observations and conversations, include a subjective quality which might potentially be impacted by individual views and interpretations. Thirdly, the research centred on the use of cross-sectional data, which offers a momentary depiction of the ergonomic analysis at a particular juncture. The use of longitudinal data will provide a more comprehensive understanding of the enduring impacts of ergonomic treatments and the dynamics of MSDs among HEMM operators. Finally, the research lacked comparison groups, such as operators employed in sectors other than mining or operators working in diverse mining contexts. A comparative study has the potential to provide valuable insights into the distinct ergonomic problems and dangers encountered by operators of HEMM in underground mining operations.

## Conclusions

The field of managing OHS in the mining industry faces challenges, including different illnesses and accidents. Currently in the Indian mining sector, there is a lack of a comprehensive and systematic OHS risk assessment model to evaluate the ergonomic-related hazards that can lead to MSDs. It is anticipated that the enhancement of OHS in the mining industry will result in a concomitant reduction in the prevalence of MSDs and accidents. This would be positively impacting worker morale, productivity, stakeholder relations, and long-term cost savings. These endeavours also aid in establishing a positive public image and may position the mine as a responsible player in the industry, thereby enhancing its long-term sustainability. The research is aimed to bridge this gap by developing a Fuzzy Inference model, specifically to address MSDs caused by ergonomic hazards for enhancing the sustainability of Indian mining industry.

The comprehensive ergonomic analysis of HEMM operators in Indian underground mines reveals the persistent presence of ergonomic hazards and their association with MSDs. The findings highlight the need for proactive ergonomic interventions and the incorporation of ergonomic principles in work process design, work tool design, and posture adoption. The introduction of fuzzy logic methods, such as the FMI, shows promise in evaluating the impact of ergonomic hazards on MSDs. The FMI results show the average MSDs score of 3.69 which states that the HEMM operators were experienced high to very high risk of MSDs. The surface plots exemplify that both work tool and work process have a greater impact on the MSDs risk in comparison to combined significance of work posture, work process and work posture and work tool. The HEMM operators were mostly affected by the lack of ergonomic work tools in the HEMM, followed by ergonomic work process and posture adoption in the work system. These results highlight the essence of evaluation of ergonomic risk which were over-shadowed in the Indian mining industry. The implementation of various initiatives such as redesign of work processes, development of ergonomic work tools, and adoption of good work postures have a significant potential for improving the safety, productivity, and general well-being of employees in underground mines.

## Data Availability

All data generated or analysed during this study are available on request from the corresponding authors.
